# The Value of Routine Lumbar Puncture

**Published:** 1920-02-28

**Authors:** 


### SYPHILIS AND THE NERVOUS SYSTEM.

### The Value of Routine Lumbar Puncture.

In any circumstances the interest attaching to the latest of the series* of published works on syphilitic infections, by Fildes and Parnell while in the Naval Service, would be considerable, but in view of the urgency with which the ultimate dangers of venereal infection are being brought to the public notice, the resulting increased attendance at venereal clinics and the great extension of these clinics themselves, a communication dealing with the frequency and date of early meningeal involvement in average cases of the disease is particularly worthy of notice and contains conclusions with which every medical officer controlling venereal treatment should immediately make himself familiar.

The present report is a direct continuation of those already published by the same authors and, as before, is based upon a large number of observations recorded upon a constant uniform system. The combined simplicity and efficiency of this process might well serve as a model to be followed in hospitals outside the confines of Service conditions, and we give the authors' own description. The italics are our own.

### An Observational Scheme.

"At Haslar every man who enters the venereal section for syphilis or suspected syphilis has the full details of his case entered upon a recording-card system, which is designed to save labour and to give all information on a systematic plan suitable for mass statistics. Every man has *all* applicable laboratory tests carried out as a routine.

"The treatment of the cases also proceeds along routine lines, particular courses being applied to cases of particular 'type.' We do not treat men as individuals but as 'types,' according to a set routine. The basis of the treatment is a series of injections of 'neo-salvarsan' compounds carried out at short intervals.

"We have further instituted a system by which it is possible (if the exigencies of the service permit) to follow the histories of all men after discharge to duty, and we hope that a sufficient number may be followed to throw important light upon the value of particular routine treatments in cases of a particular 'type.'

"We do not give accessory forms of treatment such as mercury, and endeavour to dissuade the medical officers on the ships from giving it. We thus arrange on a straight issue as to whether some five or six doses of a 'neo-salvarsan' drug are capable of curing a satisfactory percentage of men whose particular stage of the disease is met with.

"Having established this routine system of examination and treatment, we determined to investigate in a large series of cases the existence of unsuspected nervous disease, such as has been recognised to exist in cases of syphilis during recent years. We hoped that if we were able to record the outstanding features of the state of the central nervous system of *every* man, our 'follow up' system might in future throw important light upon the mechanism of the onset of the later nervous manifestation of syphilis."

Therefore have the authors instituted a system of examination of the central nervous system by colleagues specialising in the various branches concerned, and the valuable results they have already obtained give ample proof of the efficiency of their system. True it is that in trying a number of different routines there is a possible percentage of men who have not received the ideal and best treatment as is perhaps ultimately proved in their results, but we would nevertheless commend the principle when compared with the modern tendency to assume the latest and most fashionable dose-routine as necessarily the best, and to continue blindly treating along the lines it sets out. We fear this has been done at not a few of the venereal clinics developed in the last few years and is a procedure calculated to provide the minimum of knowledge for guidance of future workers, for though salvarsan may be a specific drug, we are still some distance short of a perfect knowledge of exactly how and how frequently it should be administered to produce a cure.

This particular report is, however, chiefly of interest in connection with early nervous involvement, and although the authors do not find pathological nervous changes to be quite as frequent as some earlier observers have suggested, yet they have quite clearly established that even in the earliest weeks of syphilis a patient may commonly show gross meningeal inflammation, and at the same time practically no symptoms or signs be present.

**The heaviest incidence of unsuspected nervous disease was among men who had been infected over eighteen months, and who *showed no clinical evidence of syphilis;* but a small number who were found to be affected in the earliest stages, even before the Wassermann reaction of the serum had become positive. The authors lay stress on the importance of including lumbar puncture in the routine examination of all cases of syphilis, in order that the exact nature of the conditions present may be determined before treatment is begun.**

With regard to this last recommendation we note that a great deal of cerebro-spinal fluid investigation has been done upon both the "shell-shock" and the suspected syphilitic cases now under the care of the Ministry of Pensions and the results, as far as can at present be ascertained, are largely in accordance with the Haslar findings, and have incidentally been of the greatest value in assisting a true clinical judgment of the cases to be formed.

In view, therefore, of the direct applications of this work which must in future be made to our treatment among the civil community, an outline of the essentials at least may be given. In all

* Medical Research Committee Special Report Series No. 45.

**Syphilis and the Nervous System—(*continued*).**

1,314 syphilitic men, mostly in *very early* stages, were examined, and of these 18 per cent. showed undoubted signs of an abnormal condition of the central nervous system—that is to say, 236 cases exhibited a definite excess of cells in the cerebro-spinal fluid—namely, ten or more cells to a cubic millimetre. Also the Wassermann reaction of ninety-one cases was positive in the fluid, and from the authors' table of percentages in the different "types," we may summarise by saying that an increase in cells in the cerebro-spinal fluid *does* occur in a small number of cases of primary syphilis, so early in fact that the Wassermann Reaction in the blood serum has not then become positive.

The Secondary Type.

As one passes on to the successive later stages, it is seen that during the "secondary" period twenty-one per cent. of the cases shows this increase in the cell count. In later or tertiary stages the figure is twenty-seven per cent. "It is of interest to note that type D cases with clinical signs of active syphilitic disease show a lower percentage of abnormality than cases without such signs—namely, 'latent' cases"—which is an obviously important reason for urging a far more frequent examination of the cerebro-spinal fluid than has been the custom up to quite recent times. The figures show in fact that of those men in whom more than eighteen months had elapsed since infection and yet showed no symptoms, the highest percentage was obtained—namely, thirty-four.

Of the meaning of these examinations and their bearing on the conditions of the men's nervous systems it is stated that "the nature of this abnormality in the central nervous system does not admit of doubt. It is a syphilitic meningitis. We form this opinion not only upon the presence of lymphocytes in the cerebro-spinal fluid, but also upon the frequent appearance of polymorphonuclear leucocytes in the more pronounced cases. Furthermore, in a large proportion of abnormal cases, the fluid showed a positive Wassermann reaction, while in one case we even found *S. pallida* by the method of dark ground illumination."

Cases in Practice.

If we now review the results from the aspect of the cases as they may occur to the average practitioner who examines them carefully but to some extent superficially, it seems that there must repeatedly occur, and be temporarily missed, many instances of people who have relatively active specific meningitis, but with an almost total lack of symptoms. Total lack of disability is noticed in cases with actual visible opalescence in the cerebro-spinal fluid! Also signs of a commencing retinitis may be frequently detected, but the authors note that though a certain number may show affections of the internal ear, still there is no evidence that these latter lesions are commoner than among non-syphilitics or syphilitics with intact nervous systems. In other words, the nervous extension may commence extremely early in the disease, but the only method by which it can with any certainty be either detected or its presence disproved is by examination of the thecal fluid. On the evidence they have so ably collected, it would seem that the authors are fully justified in urging that lumbar puncture should never be omitted in the routine examination of syphilis cases, whenever encountered.

In the past this simple procedure has, we fear, been far too frequently omitted upon the doubtful excuse of its possible danger. On several recent occasions we have urged that more general use should be made of it provided a few hours' complete rest immediately following can be allowed the patient. Apparently for our future anti-syphilitic treatment it is new definitely proved that cerebro-spinal fluid investigations *must* be done if any real perfection is to be attained, and certainly if a thing is worth doing at all it is worth doing well. Therefore let us act up to the Haslar teaching.

